# A DNA Adsorption-Based Biosensor for Rapid Detection of Ratoon Stunting Disease in Sugarcane

**DOI:** 10.3390/bios15080518

**Published:** 2025-08-08

**Authors:** Moutoshi Chakraborty, Shamsul Arafin Bhuiyan, Simon Strachan, Muhammad J. A. Shiddiky, Nam-Trung Nguyen, Narshone Soda, Rebecca Ford

**Affiliations:** 1 Centre for Planetary Health and Food Security (CPHFS), Nathan Campus, Griffith University, Nathan, QLD 4111, Australia; rebecca.ford@griffith.edu.au; 2 School of Environment and Science (ESC), Nathan Campus, Griffith University, Nathan, QLD 4111, Australia; simon.strachan@griffithuni.edu.au; 3 Queensland Micro- and Nanotechnology Centre (QMNC), Nathan Campus, Griffith University, Nathan, QLD 4111, Australia; sbhuiyan@sugarresearch.com.au (S.A.B.); nam-trung.nguyen@griffith.edu.au (N.-T.N.); n.soda@griffith.edu.au (N.S.); 4 Sugar Research Australia (SRA), 90 Old Cove Road, Woodford, QLD 4514, Australia; 5 Rural Health Research Institute (RHRI), Orange Campus, Charles Sturt University, Orange, NSW 2800, Australia

**Keywords:** electrochemical detection, plant pathogen diagnostic, sugarcane diseases, interfacial biosensing, resistance screening

## Abstract

Early and accurate detection of plant diseases is critical for ensuring global food security and agricultural resilience. Ratoon stunting disease (RSD), caused by the bacterium *Leifsonia xyli* subsp. *xyli* (*Lxx*), is among the most economically significant diseases of sugarcane worldwide. Its cryptic nature—characterized by an absence of visible symptoms—renders timely diagnosis particularly difficult, contributing to substantial undetected yield losses across major sugar-producing regions. Here, we report the development of a potential-induced electrochemical (EC) nanobiosensor platform for the rapid, low-cost, and field-deployable detection of *Lxx* DNA directly from crude sugarcane sap. This method eliminates the need for conventional nucleic acid extraction and thermal cycling by integrating the following: (i) a boiling lysis-based DNA release from xylem sap; (ii) sequence-specific magnetic bead-based purification of *Lxx* DNA using immobilized capture probes; and (iii) label-free electrochemical detection using a potential-driven DNA adsorption sensing platform. The biosensor shows exceptional analytical performance, achieving a detection limit of 10 cells/µL with a broad dynamic range spanning from 10^5^ to 1 copy/µL (r = 0.99) and high reproducibility (SD < 5%, n = 3). Field validation using genetically diverse sugarcane cultivars from an inoculated trial demonstrated a strong correlation between biosensor signals and known disease resistance ratings. Quantitative results from the EC biosensor also showed a robust correlation with qPCR data (r = 0.84, n = 10, *p* < 0.001), confirming diagnostic accuracy. This first-in-class EC nanobiosensor for RSD represents a major technological advance over existing methods by offering a cost-effective, equipment-free, and scalable solution suitable for on-site deployment by non-specialist users. Beyond sugarcane, the modular nature of this detection platform opens up opportunities for multiplexed detection of plant pathogens, making it a transformative tool for early disease surveillance, precision agriculture, and biosecurity monitoring. This work lays the foundation for the development of a universal point-of-care platform for managing plant and crop diseases, supporting sustainable agriculture and global food resilience in the face of climate and pathogen threats.

## 1. Introduction 

Ratoon stunting disease (RSD) remains one of the most insidious and economically damaging diseases affecting sugarcane production globally. First identified in Queensland, Australia, in the mid-1940s in the Q28 sugarcane cultivar [1], RSD can cause significant yield reductions of 12–37% under normal conditions and up to 60% in drought-stressed crops, while also compromising varietal quality [2,3,4]. The causative agent, *Leifsonia xyli* subsp. *xyli* (*Lxx*) is a xylem-limited, slow-growing bacterium [5,6] that causes non-specific physiological symptoms—such as reduced tillering, stalk diameter, and plant height—making RSD visually indistinguishable from drought or poor agronomic practices. This diagnostic ambiguity leads to unchecked propagation of *Lxx* through infected planting materials and farm machinery, contributing to widespread and persistent disease transmission [7].

The minute size (195–220 nm diameter) and fastidious nature of *Lxx* present considerable challenges in terms of isolation and culture [8,9]. As a result, various serological [10,11] and molecular detection platforms—including PCR [12,13], multiplex PCR [14], nested PCR [15], real-time PCR [16,17], and loop-mediated isothermal amplification (LAMP) [18]—have been developed. However, these tools remain restricted to centralized laboratories due to their dependence on sophisticated equipment, labor-intensive DNA extraction steps, and reliance on commercial kits or complex chemical reagents [18,19,20]. Diagnostic delays, logistical barriers, and high operational costs continue to limit widespread adoption, especially in remote or resource-limited farming contexts. To address this gap, we introduce a novel amplification-free electrochemical (EC) nanobiosensor platform that leverages potential-induced affinity (adsorption) between the gold surface and nucleic acid for direct detection of *Lxx* DNA from sugarcane xylem sap. This platform integrates a one-step, boiling-based DNA lysis with sequence-specific magnetic capture and label-free EC signal readout. Unlike existing molecular assays, our method eliminates the need for column- or solvent-based nucleic acid extraction; it employs a sequence-specific magnetic bead capture step, which constitutes a non-conventional yet effective form of nucleic acid purification. It also avoids enzymatic amplification and specialized laboratory infrastructure, offering a portable, low-cost, and rapid diagnostic solution suitable for on-farm deployment by non-specialist users. While affinity interactions-based EC DNA sensors have shown promise for pathogen detection in medical diagnostics [21,22,23,24,25,26], their application in plant pathogen diagnostics remains in its infancy [26,27,28,29,30]. Recent advances in electrochemical biosensing have established DNA-based detection platforms as powerful tools for the rapid and sensitive identification of plant pathogens. These methods typically involve probe-functionalized electrodes coupled with redox-active indicators to convert nucleic acid recognition events into quantifiable electrochemical signals. For instance, methylene blue-labeled probes have enabled sensitive detection of bacterial and fungal pathogens through signal amplification upon hybridization [26,27,28]. In parallel, label-free strategies have gained traction, leveraging changes in charge transfer resistance or suppression of redox current resulting from DNA adsorption at the electrode interface [29,30]. Collectively, these studies underscore the expanding potential of electrochemical DNA sensors for field-deployable, amplification-free diagnostics and provide a strong conceptual framework for the approach presented in this work. To our knowledge, this is the first report of an EC nanobiosensor designed for the direct, amplification-free detection of *Lxx* DNA from plant samples, achieving high sensitivity, quantitative resolution, and excellent correlation with qPCR. Importantly, this method represents more than a disease-specific diagnostic—it establishes a generalizable biosensing framework for early detection of diverse plant pathogens. While it was developed to detect *Leifsonia xyli* subsp. *xyli* in sugarcane, the same underlying approach could be adapted to detect other plant pathogens (bacterial, viral, or fungal) in other crops, just by changing the DNA probe sequence. As such, it offers immense potential as a platform technology for crop health monitoring, biosecurity surveillance, and climate-resilient agriculture. By enabling rapid identification and intervention, our biosensor contributes directly to enhancing food security, reducing yield losses, and supporting sustainable agricultural practices in the face of increasing pathogen pressure and environmental stress.

## 2. Materials and Methods

### 2.1. Reagents and Materials

Screen-printed gold electrodes (SPGEs, DRP-220AT) with integrated three-electrode systems were obtained from Metrohm Dropsens (Llanera, Spain). Gold (III) chloride trihydrate, synthetic DNA sequences, and primers (Supplementary Table S1) were purchased from Sigma Aldrich (Castle Hill, Australia) and Integrated DNA Technologies (Coralville, IA, USA), respectively. UltraPure^TM^ DNase/RNase-free distilled water was from Invitrogen (Melbourne, Australia). Electrochemical measurements were carried out using a CH1040C potentiostat (CH Instruments, Inc., Austin, TX, USA).

### 2.2. Source of Inoculum and Culture Conditions 

*Lxx* was isolated from infected sugarcane stalks at Sugar Research Australia (SRA), Woodford, following sap extraction using positive pressure methods as described in Ngo et al. [11]. Sap was filtered (0.2 µm), inoculated into modified S8 medium, and cultured at 28 °C in the dark for four weeks [5,19]. Presence of *Lxx* was confirmed by qPCR. Negative controls included *Xanthomonas albilineans* (*Xalb*) and *Ceratocystis paradoxa* (*Cpar*), cultured using standard methods [31,32].

### 2.3. Field Trial and Sample Collection

A randomized complete block field trial was established at SRA Woodford (S 26.93°, E 152.78°) in September 2020, using 10 sugarcane cultivars with known RSD resistance ratings (CP72-2086, Ho06-537, Q232, Q253, SRA20, Q208, WSRA24, Q242, SRA26, and SRA22) as shown in Table 1 [11,33,34,35]. One-budded setts were hot-water treated (52 °C, 30 min), inoculated with *Lxx* [35], germinated in moistened vermiculite, and transplanted to the field. At 53 weeks post-inoculation, xylem sap was extracted from three stalks per cultivar and stored at –20 °C until use.

### 2.4. DNA Isolation

For electrochemical analysis, rapid and simple DNA isolation was achieved by boiling sap spiked with serially diluted *Lxx* cultures or field samples (95 °C, 2 min), as previously described [21,22]. Supernatants were directly used as templates (10 µL per assay). For qPCR, genomic DNA was extracted using the PureLinkTM Microbiome DNA kit (Thermo Fisher Scientific, Scoresby, VIC, Australia) following the manufacturer’s instructions. All assays were performed in triplicate and repeated independently three times.

### 2.5. Target Selection and Primer Design 

A 34 bp region within the 16S–23S intergenic spacer (IGS) of *Lxx* (GenBank: AE016822.1) was targeted using a capture probe (*Lxx*CP1) [36,37,38]. Primers for qPCR (*Lxx*_EC_FP/RP) targeting a 133 bp segment within the same region were designed using NCBI Primer-BLAST [39] and validated for specificity using BLASTn [40]. Structural integrity of oligonucleotides was assessed using OligoAnalyzerTM (Integrated DNA Technologies, Inc., Coralville, IA, USA).

### 2.6. Probe Hybridization and Magnetic Isolation

Target DNA was captured using sequence-specific magnetic beads following previously established protocols [41,42]. Purified products were stored at −20 °C for subsequent EC quantification. All experiments included triplicate biological and technical replicates.

### 2.7. Sensor Fabrication and Assay Optimization

To enhance performance, the screen-printed gold electrode (SPGE) surfaces were pretreated and modified with gold nanoparticles (AuNPs) following the protocol described by Zhang et al. [43]. Briefly, a droplet of gold nanoparticle solution was applied to the working electrode surface, and electrochemical deposition was carried out by applying a potential of 0.2 V (vs. Ag/AgCl) to ensure uniform immobilization of the nanoparticles onto the gold surface. The electroactive surface area was calculated using the Randles–Sevcik equation [44]. Target DNA was deposited onto the SPGEs by applying a constant potential of 0.6 V (vs. Ag/AgCl) for 60 s to adsorb the DNA onto the electrode surface. The deposition conditions (potential and time) were optimized to achieve maximum adsorption while minimizing nonspecific binding, based on previous studies [45,46]. After deposition, the electrodes were washed three times with 10 mM PBS.

### 2.8. Electrochemical Detection

Differential pulse voltammetry (DPV) was performed in 2 mM [Fe(CN)_6_]^3−^ solution (–0.2 V to 0.45 V; amplitude 0.05 V) to quantify DNA adsorption. The current response was normalized using the following equation:

$$=[(i_{Bare}-i_{Adsorbed})/i_{Bare}]\times 100$$

where *i_Bare_* and *i_Adsorbed_* represent current densities before and after DNA deposition, respectively.

### 2.9. qPCR Validation and Gel Electrophoresis

Quantitative PCR was conducted using a CFX96 Touch system (Bio-Rad Laboratories, Inc., Gladesville, NSW, Australia) with reaction conditions per manufacturer protocol (New England Biolabs, Inc., Ipswich, MA, USA): 98 °C (15 s), 52 °C (30 s), and 72 °C (30 s) for 40 cycles. Positive results were defined as Cq < 40 or undetermined. qPCR standard curves were generated from serially diluted *Lxx* DNA (10^5^–10^0^ cells/µL) in spiked sap. Gel electrophoresis was performed on 1% agarose gels and visualized using SYBR Safe DNA stain under UV illumination (90 V, 40 min), with 133 bp amplicons visualized using GeneRuler ladder (Thermo Fisher Scientific, Scoresby, VIC, Australia).

### 2.10. Statistical Analysis

All data are presented as mean ± standard error (n = 3). No statistical LoD (e.g., 3*σ*/*s*, where *σ* is the standard deviation of the blank and *s* is the slope of the calibration curve) was calculated; instead, the lowest detectable concentration was used based on the experimentally observed signal distinguishability from the blank. Statistical analyses and visualizations were performed using OriginPro v9.9.0.225 (OriginLab, Northampton, MA, USA), Microsoft Excel 365, and RStudio v4.2.3 [47]. Correlations between EC and qPCR results were assessed using Spearman’s rank correlation. Figures were created using BioRender [48], SnapGene (www.snapgene.com), and Microsoft PowerPoint 365.

## 3. Results and Discussion

### 3.1. Assay Design 

**Figure 1:**
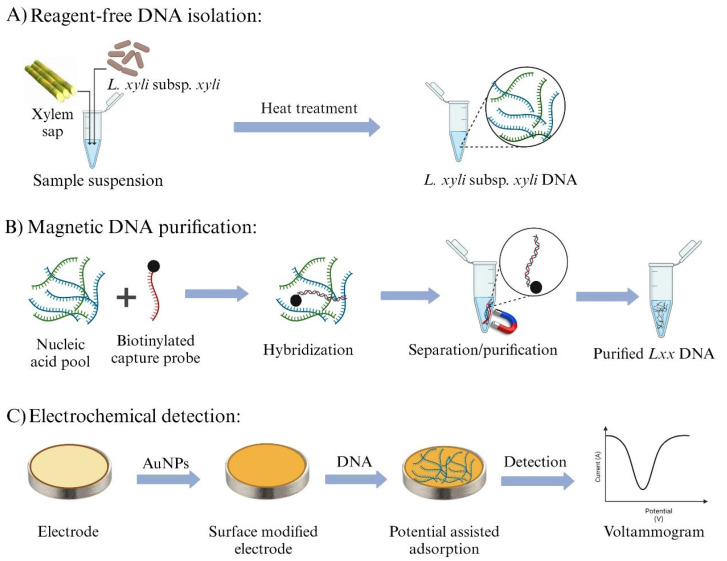
Overview of the electrochemical assay workflow for detecting *Lxx* DNA. (**A**) Bacterial DNA is isolated from sugarcane xylem sap using a simple boiling lysis method. (**B**) The isolated *Lxx* DNA is selectively captured using biotinylated complementary DNA probes and isolated via streptavidin-coated magnetic beads. (**C**) Captured *Lxx* DNA is thermally released and field-induced to adsorb onto AuNPs-modified SPGE, followed by electrochemical detection using DPV.

Figure 1 presents the outline of the newly developed *Lxx* detection assay. The assay begins with the release of bacterial DNA from sugarcane xylem sap using a simple boiling lysis technique, followed by direct capture of *Lxx* target DNA through a complementary biotinylated probe (*Lxx*CP1) immobilized on streptavidin-coated magnetic beads (Figure 1A). The captured *Lxx* targets were magnetically purified through multiple washing steps to remove non-target molecules while retaining the nanoparticle/probe/target complexes. Subsequently, heat treatment was applied to dissociate the *Lxx* targets from the capture probe/nanoparticle complexes, allowing the probe-bound target sequences to be released into solution. The released *Lxx* targets were then adsorbed onto the AuNPs-modified SPGE surface via a potential-induced gold–DNA affinity interaction (Figure 1B). Finally, electrochemical detection was performed by voltammetric interrogation using [Fe(CN) _6_]^3−^, generating a differential pulse voltammetry (DPV) readout to quantify the captured target (Figure 1C). Adsorption of *Lxx* DNA on the electrode surface reduces the electrochemical signal due to increased coulombic repulsion between the negatively charged ferricyanide ions and the electrode surface.

### 3.2. Assay Optimization

To enhance assay sensitivity, the SPGE surface was modified with AuNPs. This modification resulted in a twofold increase in current density response (mean current density 8.54 vs. 4.12 mA cm^−2^; n = 3) (Figure S1). This enhancement aligns with prior reports where AuNPs, owing to their high conductivity, facilitate rapid electron transfer between the electrolyte solution and the transducer [49,50,51,52,53,54,55,56]. The modification reduces the surface impedance of the working electrode, enabling detection of minute changes in electron transfer at the electrode–solution interface [54]. Furthermore, this approach leverages the high biocompatibility of AuNPs, which helps preserve the biological activity of immobilized molecules over extended periods [57,58,59]. The AuNP-modified electrodes in this study were fabricated using a well-established electrochemical deposition method, as described by Zhang et al. [43]. Although direct morphological characterization via SEM or TEM was not conducted, this protocol has been extensively validated in the literature for producing uniform, nanoscale gold coatings with high electrochemical activity. The significant enhancement in current response and sensitivity observed in our system (Figure S1) is consistent with effective AuNP deposition and corroborates previous reports employing similar fabrication strategies. This method was selected for its simplicity, scalability, and reproducibility. Nonetheless, we acknowledge the value of direct structural validation, and future studies will incorporate SEM/TEM imaging to further confirm nanoparticle morphology and surface distribution.

For improved target binding specificity and quantification accuracy, the optimal potential and deposition time for *Lxx* DNA adsorption were determined to be +600 mV and 60 s, respectively (Figures S2 and S3). The electrochemical signal intensity is directly dependent on the amount of DNA adsorbed onto the electrode surface. Electrodes with low DNA surface coverage exhibited electrochemical signals comparable to those of unmodified electrodes, as ferricyanide ions could readily access the surface, resulting in reduced coulombic repulsion and an increased faradaic current. Therefore, maximizing DNA adsorption under optimized conditions is critical for achieving high assay sensitivity and rapid response [45,46,60].

**Figure 2:**
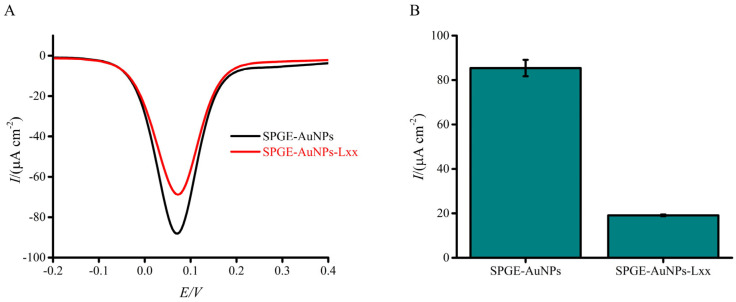
Electrochemical response of the assay to the presence or absence of synthetic *Lxx* target DNA (10 pM). (**A**) DPV signals show a clear reduction in current in the presence of the target due to DNA adsorption at the electrode surface. (**B**) Bar graph showing the mean percentage change in current density relative to the blank (no target) condition. Error bars represent standard deviations from independent runs with separately prepared electrodes (n = 3).

Assay functionality was evaluated by comparing electrochemical responses in the presence and absence of synthetic *Lxx* targets (Figure 2A,B). Figure 2B illustrates the calculated percentage change in current density between the blank (no DNA) and test conditions. This normalization emphasizes the relative suppression in electrochemical signal due to target DNA adsorption. The presence of targets caused a significant decrease in current response compared to the no-target control, demonstrating the assay’s effectiveness. This decrease arises from the combined effects of (i) coulombic repulsion between the negatively charged adsorbed DNA and the ferricyanide ions at the AuNP-modified SPGE gold sensor surface via the DNA–gold affinity interaction and (ii) steric hindrance of the active sites—some of which are already occupied by adsorbed DNA—preventing redox species from contacting the electrode surface. These repulsion and steric hindrance effects impede electron transfer during the one-electron redox reaction, leading to a diminished faradaic current relative to the control [61]. In addition to steric hindrance and electrostatic repulsion, it is plausible that DNA adsorption induces interfacial changes, such as conformational rearrangements or partial hybridization-like interactions that modulate the charge transfer resistance at the electrode surface. Prior studies have shown that DNA hybridization can significantly alter electrode impedance, thereby affecting redox probe access and overall signal output [43]. Although this study focused on potential-induced adsorption of single-stranded DNA and did not involve classical hybridization events, such impedance-related effects cannot be entirely ruled out. While electrochemical impedance spectroscopy (EIS) was not performed here, the consistent trend in current suppression with increasing DNA concentration, combined with high reproducibility and strong linearity (R^2^ = 0.98), supports our hypothesis that the signal decreases are due to DNA blocking the electrode surface and repelling charged molecules. Future studies incorporating EIS measurements will be valuable for dissecting the contributions of impedance modulation alongside steric and electrostatic effects, thereby offering deeper mechanistic insight into DNA-induced signal variation.

### 3.3. Assay Sensitivity and Specificity

**Figure 3:**
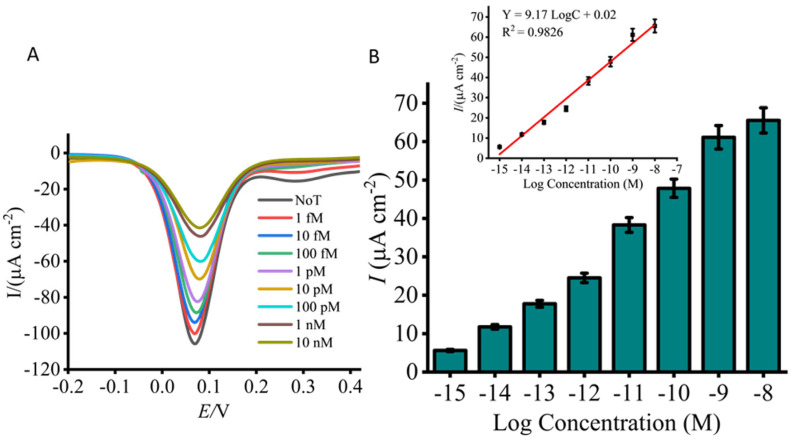
Analytical sensitivity of the assay using titrated concentrations of synthetic *Lxx* target DNA (1 nM to 1 fM). (**A**) DPV signals reveal concentration-dependent decreases in current density relative to the no-target control (NTC). (**B**) Bar graph summarizes the signal changes across tested concentrations. The inset shows the calibration curve (log concentration vs. current density), indicating strong linearity across the tested range. Error bars represent standard deviations (SD < 5%) from independent runs with separately prepared electrodes (n = 3).

The sensitivity of the developed *Lxx* assay was initially evaluated using a titration series of synthetic *Lxx* targets spiked into fresh, uncontaminated sugarcane sap (Figure 3A,B). As the concentration of *Lxx* increased, a corresponding decrease in the DPV current signal was observed, attributed to a greater number of target DNA species adsorbing on the sensor surface [62]. This increased adsorption enhanced the repulsion and steric hindrance effects of approaching [Fe(CN)_6_]^3−^ ions, hindering their diffusion to the electrode surface and thereby reducing the faradaic current. The detection limit was assessed over a concentration range spanning from 10 nM to 1 fM, and the resulting calibration plot demonstrated excellent linearity. The linear regression equation was y = 9.17 logC + 0.02 (where y represents the change in current and C the concentration of *Lxx*), with a correlation coefficient (r) of 0.9826 (Figure 3B inset). Compared to the no-template control (NTC), which exhibited a high current response due to an unblocked sensor surface, the assay achieved a detection limit down to 1 fM with a reproducibility of %SD ≤ 5% (n = 3). It is acknowledged that minor variations in current density values, particularly for the blank and 10 pM DNA samples, are observed between Figure 2 and Figure 3. These figures represent results from independent experimental runs, each conducted using separately fabricated screen-printed electrodes and freshly prepared reagents. Such variations are expected in electrochemical biosensing systems and are primarily attributed to inherent differences in electrode surface morphology, ink composition, and handling of biological samples. Despite these run-to-run variations, the data exhibits high internal consistency within each experiment. Specifically, the standard deviation across replicates remained consistently low (SD < 5%, n = 3), reflecting excellent repeatability under identical conditions. Furthermore, the platform demonstrated a strong and reproducible linear relationship between target DNA concentration and electrochemical response (R^2^ = 0.98), confirming its analytical reliability.

Our assay exhibited sensitivity surpassing previous sugarcane pathogen diagnostic platforms by factors of 100 and 1000, outperforming detection limits reported at 100 fM and 10 pM by Umer et al. [63] and Wongkaew and Poosittisak [28], respectively. In contrast, a recent AuNP-based plant virus diagnostic platform reported by Khater et al. [57] achieved a detection limit of only 100 nM, with a logarithmic response limited to a narrow concentration range (0.1–10 mM). Notably, the platform developed by Siddiquee et al. [64] demonstrated an attomolar-level limit of detection across an extensive dynamic range (1.0 × 10^−18^ to 1.82 × 10^−4^ mol L^−1^). While this approach surpassed the detection limit of our assay, it involved complex sensor fabrication and a labor-intensive phenol-chloroform DNA extraction process. In contrast, our assay offers a straightforward, cost-effective, and quantitative method, employing a simple, field-applicable DNA isolation technique and eliminating the need for complex sensor fabrication. Furthermore, our assay does not require target amplification, labeling, or antibodies. To contextualize the performance of our label-free electrochemical biosensor, a comparison was made with selected electrochemical DNA sensing platforms employing redox reporters such as methylene blue or ferrocene. As shown in Table 2, our method shows comparable or superior sensitivity, a broader dynamic range, and significantly reduced assay time—achieved without the need for probe labeling or amplification steps. This underscores the potential advantages of our approach for rapid and field-deployable diagnostics.

**Figure 4:**
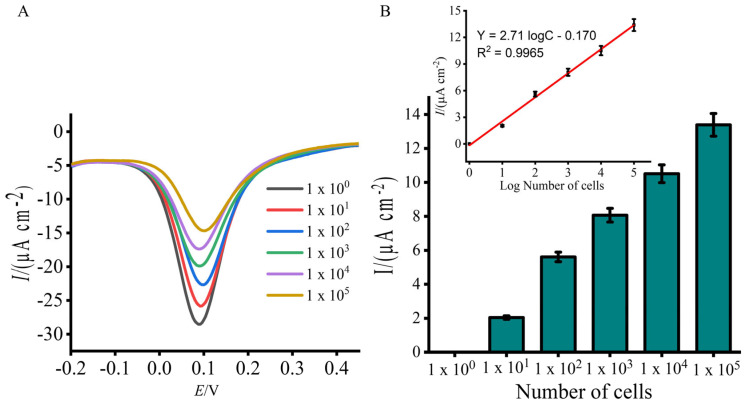
Sensitivity evaluation using total DNA extracted from known numbers of *Lxx* cells (10^5^ to 10 cells/μL). (**A**) DPV signals show decreasing current with increasing input cell numbers, corresponding to higher DNA loads. (**B**) Mean percentage current response change for each input cell concentration. Inset displays a linear calibration curve for DNA quantity vs. EC signal. Error bars show SD from three replicates.

Achieving a broad detection range and establishing a quantitative relationship between the electrochemical signal and *Lxx* concentration are crucial for accurate quantification. To assess this, bacterial samples were spiked with known *Lxx* cell concentrations (10^5^ to 10^0^ cells/μL) in fresh, uncontaminated sugarcane sap, and electrochemical detection was performed. The DPV responses exhibited a robust linear correlation across the tested concentration range (Figure 4A,B). Compared to the NTC, a significant decrease in current was observed with increasing target concentrations, confirming the assay’s capability to detect *Lxx* sequences. Moreover, the assay showed a dynamic range spanning five orders of magnitude, enabling quantification of *Lxx* in sap samples with widely varying pathogen loads. The assay detected *Lxx* at concentrations as low as 10 cells (Figure 4A,B), establishing a simple and robust diagnostic method that compares favorably with other reported electrochemical methods [29,30]. While minor variations were observed between replicate experiments using different electrode batches, all measurements showed high consistency within replicates (SD < 5%, n = 3), indicating good intra-assay reproducibility. Such variability is typical in biosensor systems involving biological samples and surface-adsorbed layers.

**Figure 5:**
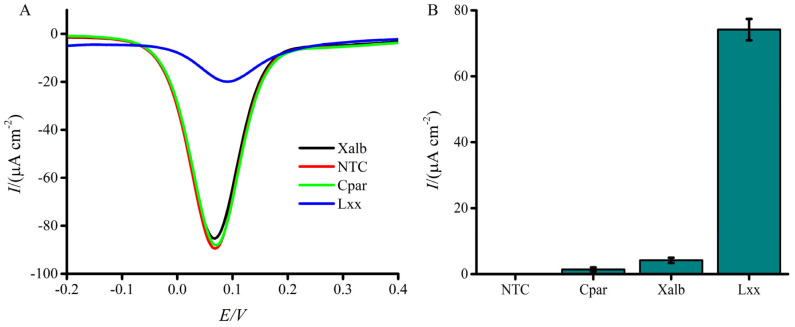
Specificity of the electrochemical assay for *Lxx* detection. (**A**) DPV signals compare current responses to DNA from *Lxx*, Xanthomonas albilineans (*Xalb*), and *Ceratocystis paradoxa* (*Cpar*) cells. (**B**) Bar graph illustrates the relative percentage change in current response for each pathogen. A significantly higher signal suppression is observed with *Lxx* compared to other organisms. NTC = no target control; NG1 = *Xalb* (negative control 1); NG2 = *Cpar* (negative control 2). Error bars indicate SD (n = 3).

Specificity was evaluated using 10^3^ cells/µL of other sugarcane pathogen contaminants, *Xalb* and *Cpar*. Only marginal decreases in current response (1.39 and 4.18 µA, respectively) were observed relative to the NTC (Figure 5A,B), indicating strong specificity for *Lxx*. By comparison, the presence of *Lxx* resulted in a twofold lower current response than the no-target control (7.42 µA) (Figure 5B). These results indicate that the proposed biosensor possesses promising specificity for *Lxx* detection and holds considerable potential for biological applications. While a full titration of non-target DNA was not conducted, the minimal signal suppression observed in the specificity test (Figure 5B) suggests that increasing concentrations of non-target DNA would not yield significant changes in current response due to the lack of probe–target complementarity.

Reproducibility was confirmed by a relative standard deviation of electrode measurements below 5% (n = 3), demonstrating acceptable assay consistency. Conventional nucleic acid biosensors rely on the specific binding of target sequences to complementary probes immobilized on a two-dimensional transducer surface, which can suffer from interference by non-specific molecules. In contrast, our approach employs selective capture of *Lxx* DNA via complementary probes followed by magnetic bead-based isolation of the target DNA. The magnetic washing and purification steps effectively remove matrix effects and substantially reduce non-specific interferences, enhancing assay robustness. Reproducibility was evaluated by performing triplicate measurements (n = 3) at each DNA concentration using independently prepared AuNP-modified electrodes. The standard deviation (SD) was calculated for each concentration point and found to be <5%, indicating high reproducibility under the described operating conditions. While this study focused on demonstrating the sensing mechanism and analytical performance, we acknowledge that sensor stability, storage conditions, and long-term usability were not evaluated and remain important areas for future investigation to support real-world deployment.

Although this study did not include direct surface characterization techniques such as XPS or FTIR to confirm DNA adsorption onto the AuNP-modified SPGE surface, the electrochemical response patterns observed in this work strongly support adsorption-based interactions. The consistent and concentration-dependent decrease in current density following DNA deposition (Figure 2 and Figure 3), coupled with the linear correlation between target DNA concentration and signal suppression (R^2^ = 0.98), is well-aligned with prior studies utilizing potential-induced DNA adsorption on gold surfaces [21,22,23,24,25,26,45,46]. These findings, along with the use of a well-established adsorption potential (+600 mV), support the proposed sensing mechanism. Nevertheless, we acknowledge this as a limitation and propose that future work includes surface analytical techniques (e.g., XPS or AFM) to provide direct validation of the adsorption process.

### 3.4. Detection of Lxx in Sugarcane Xylem Sap Samples

**Figure 6:**
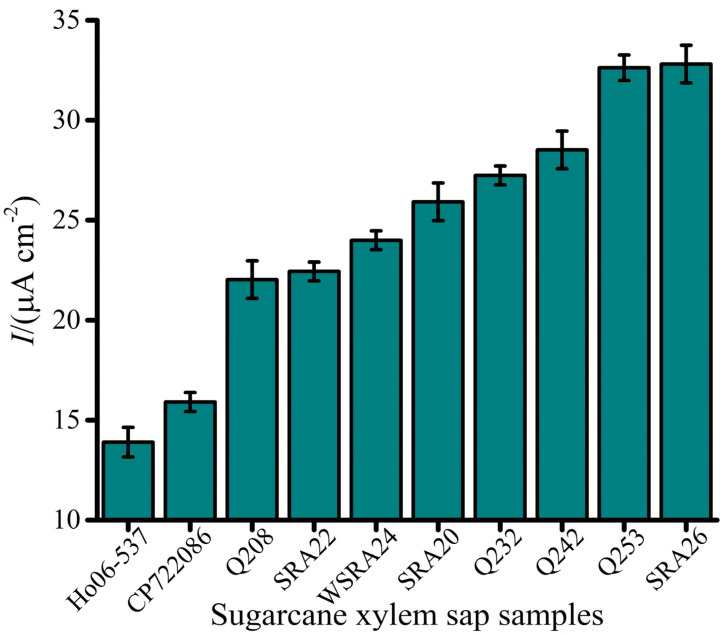
Electrochemical detection of *Lxx* in sugarcane xylem sap samples collected from field trials. Bar graph displays current density values for multiple sugarcane cultivars tested during SRA Woodford RSD screening trials. Variations in EC signal correlate with bacterial load and cultivar susceptibility. Error bars represent SD of triplicate measurements.

The developed *Lxx* electrochemical biosensor was applied to detect varying amounts of *Lxx* in xylem sap samples from 10 sugarcane cultivars collected from a field trial. Detected *Lxx* DNA levels in most samples fell within the assay’s detection limit of 1 fM, highlighting its suitability for real-world analysis. Detected *Lxx* levels corresponded well with cultivar disease ratings. For example, samples from cultivars Ho06-537 and CP72-2086 exhibited relatively low current responses, indicative of lower bacterial loads and consistent with their moderate resistance to RSD (Figure 6). Cultivars Q208 and SRA22 were classified as intermediately resistant, while WSRA24, SRA20, and Q232 were considered susceptible. Intermediate susceptible cultivars Q242, Q253, and SRA26 were identified as highly susceptible (Figure 6). These results aligned closely with established industry disease ratings [35].

### 3.5. Validation with qPCR

**Figure 7:**
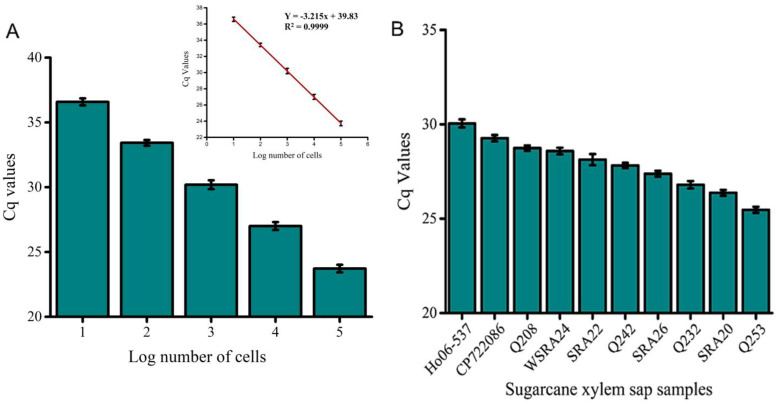
qPCR validation of the electrochemical assay. (**A**) Absolute quantification of *Lxx* DNA in known cell concentrations (10^5^ to 10 cells/µL) confirms the sensitivity of the assay. (**B**) Quantitative detection of *Lxx* in selected field samples infected with RSD. qPCR data show good agreement with EC assay trends. Error bars indicate SD across three independent experiments.

A strong correlation was observed between electrochemical and qPCR quantification, confirming the biosensor’s suitability for *Lxx* detection (Figure 7). qPCR effectively amplified the target intergenic spacer (IGS) region from DNA extracted from 100 cells, with clear and distinct amplicon bands observed via gel electrophoresis (Figure S4). No amplification or bands were detected in the NTC samples. The strong correlation (r = 0.84, *p* < 0.001; Table 3) between the EC signal and qPCR quantification underscores the assay’s reliability. Notably, the electrochemical assay demonstrated a 10-fold greater sensitivity compared to qPCR (Figure 4 vs. Figure 7A).

For field sample validation, qPCR successfully amplified the target region across all analyzed samples with varying cycle thresholds, reflecting differences in pathogen load, susceptibility, and detection limit (Figure 7B). This trend was consistent with the electrochemical assay results (Figure 6). All analyzed samples exhibited clear qPCR amplicon bands (Figure S5), while NTCs showed no amplification. The qPCR cycle threshold (Cq) values and electrochemical signals exhibited negative correlations with RSD resistance ratings (r = −0.78 and −0.87, respectively). Furthermore, qPCR and electrochemical values showed a strong positive correlation (r = 0.87, *p* < 0.001) (Table 3). The strong agreement between electrochemical current responses and qPCR Cq values (r = 0.84, *p* < 0.001) confirms the assay’s robustness for detecting and quantifying *Lxx* in xylem sap samples. Negative control samples exhibited EC signals comparable to background levels, with current changes below 10%, confirming assay specificity. Successful application of the biosensor to real biological samples (Figure 6), coupled with validation through qPCR and gel electrophoresis (Figure 7 and  Figure S5), underscores the technique’s potential for practical biological diagnostics. Although slight variability in Cq values was observed at the lowest target concentration (10 cells/µL), the replicates remained consistent with acceptable technical variation. The negative slope confirms the expected inverse relationship between Cq and template quantity. A summary comparison of our biosensor with conventional nucleic acid amplification and hybridization-based techniques is provided in Table 4. This highlights the potential field-deployable advantages of our approach in terms of speed and simplicity.

## 4. Conclusions 

In this work, we present a proof-of-concept electrochemical assay capable of detecting *Lxx*, the causative agent of ratoon stunting disease in sugarcane, with ultrahigh sensitivity and excellent analytical performance. The assay enabled quantitative detection of *Lxx* DNA at concentrations as low as 1 fM, with a wide dynamic range (1 fM to 10 nM), strong linearity (r = 0.99), and high reproducibility (SD < 5%, n = 3). Key features of this platform include its compatibility with a simplified boiling lysis DNA extraction protocol, eliminating the need for labor-intensive or kit-based methods, and its use of low-cost, disposable SPGE (~US$5 per sensor), which brings the total assay cost to under US$10 per sample. Notably, the sensor requires no complex surface modification or amplification steps, distinguishing it from many existing biosensing platforms. Beyond sensitivity and cost-effectiveness, the modular design of this assay provides a highly adaptable framework for broader applications. By tailoring the capture probes, the platform could be readily extended to detect a range of plant pathogens in diverse sample matrices, including soil, sap, or leaf extracts. The integration of multi-well electrodes further enables potential high-throughput or multiplexed analyses. Taken together, this study lays the foundation for the development of field-deployable diagnostic devices for on-site agricultural pathogen monitoring. With future integration into automated or smartphone-assisted platforms, this assay could support rapid disease surveillance, geospatial risk mapping, and early warning systems for exotic pathogens, especially at biosecurity checkpoints such as ports and border entries. This approach contributes to the growing need for portable, sensitive, and cost-effective diagnostics in sustainable agriculture and plant biosecurity. In summary, this work presents a simple, rapid, and label-free electrochemical biosensor for the detection of *Leifsonia xyli* subsp. *xyli*, demonstrating high sensitivity, minimal sample preparation, and suitability for field application. While the electrochemical data strongly support DNA adsorption onto the SPGE surface, future studies could incorporate complementary surface analytical techniques, such as XPS, atomic force microscopy (AFM), or quartz crystal microbalance (QCM), to directly visualize and chemically characterize the DNA layer. Further mechanistic investigations using electrochemical impedance spectroscopy (EIS) can also help distinguish between physical adsorption and DNA hybridization-induced impedance changes at the electrode interface. Most importantly, systematic studies are required to assess the sensor’s stability and shelf life under conditions relevant to real-world field applications.

## Figures and Tables

**Table 1:** List of sugarcane varieties and their ratoon stunting disease resistance rating and rating categories (Ngo et al. [35]).

Variety	RSD Rating	Rating Category
Q253	8	Susceptible
SRA26	6	Intermediate susceptible
Q242	7	Susceptible
Q232	6	Intermediate susceptible
SRA20	8	Susceptible
WSRA24	4	Intermediate resistant
SRA22	3	Moderately resistant
Q208	5	Intermediate resistant
CP72-2086	3	Moderately resistant
Ho06-537	3	Moderately resistant

**Table 2:** Comparative analysis of electrochemical DNA biosensors using redox reporters (e.g., methylene blue, and ferrocene) vs. the proposed label-free platform.

Method	Label	Detection Limit	Dynamic Range	Assay Time	Reference
This work	None	1 fM	1 fM–10 nM	~30 min	This work
MB-modified probe	Methylene blue	10 fM	10 fM–1 nM	~1.5 h	[28]
Ferrocene–DNA hybrid sensor	Ferrocene	50 fM	50 fM–5 nM	~2 h	[29]
MB + LAMP	Methylene blue	1 fM	1 fM–100 pM	~90 min	[28]
Fc-DNA on gold nanocomposite	Ferrocene	100 fM	100 fM–10 nM	~1.5 h	[57]

**Table 3:** Spearman correlation coefficients using Cq Values of EC and qPCR methods for comparing RSD resistance ratings among ten sugarcane varieties.

Factors *^a^*	qPCR	Resistance Rating
EC *^b^*	0.84 **	−0.78 *
qPCR *^c^*		−0.87 **

*^a^* Correlations were based on 10 observations, * = significant at or < 0.05 levels, ** = significant at or <0.001. *^b^* EC = EC data from isolated DNA of xylem sap samples by heat induced cell lysis. *^c^* qPCR = qPCR data from commercial kit-based DNA extraction of xylem sap samples.

**Table 4:** Comparison of the proposed electrochemical biosensor with conventional nucleic acid extraction and detection methods.

Methods	Nucleic Acid Extraction	Detection Limit	Sample Preparation-to-Result	Reference
This study (EC biosensor)	Boiling (2 min)	10 cells/µL or 1 fM	~30 min	This work
Conventional PCR	Commercial kit	~10^3^–10^4^ cells/µL	4–5 h	[12,13]
Real-time PCR (qPCR)	Commercial kit	~100 cells/µL	3–5 h	[16,17]
Nested PCR	Commercial kit	~10 cells/µL	4–6 h	[15]
LAMP	Commercial kit or crude	~10–100 cells/µL	60–90 min	[18]
Dot Blot/Hybridization	Commercial kit or crude	~10^4^–10^5^ cells/µL	6–8 h	[10]

## Data Availability

Data will be made available upon request.

## Supplementary Materials

The following supporting information can be downloaded at: https://www.mdpi.com/article/10.3390/bios15080518/s1.

## Abbreviations

RSD	Ratoon Stunting Disease
*Lxx*	*Leifsonia xyli* subsp. *xyli*
Xalb	Xanthomonas albilineans
Cpar	Ceratocystis paradoxa
PCR	Polymerase Chain Reaction
qPCR	Quantitative Polymerase Chain Reaction
LAMP	Loop-Mediated Isothermal Amplification
EC	Eectrochemical
DNA	Deoxyribonucleic Acid
RNA	Ribonucleic Acid
SPGE	Screen-Printed Gold Electrode
PDA	Potato Dextrose Agar
RCB	Randomized Complete Block
mL	Milliliter
EC	Electrochemical
Bp	Base pair
IGS	Intergenic Spacer
NCBI	National Center for Biotechnology Information
BLASTn	Basic Local Alignment Search Tool for nucleotides
[Fe(CN)^6^]^3–^	Ferricyanide ion
cm^2^	Square centimeter
Fe^3+^	Ferric ion
Fe^2+^	Ferrous ion
n	Number
s^−1^	Per second
Vs^−1^	Volts per second
mol cm^−3^	Mole per cubic centimeter
Ag/AgCl	Silver/Silver Chloride
mV	Millivolts
AuNPs	Gold Nanoparticles
CV	Cyclic Voltammetry
DPV	Differential Pulse Voltammetry
mM	Millimolar
V	Volt
i	Current
Cq	Cycle Quantification
µL	Microliter
SD	Standard Deviation
r	Correlation coefficient
TAE	Tris-Acetate-EDTA
*Lxx*CP1	*Lxx*-specific Capture probe-1
mA cm^−2^	Milliamps per square centimeter
nM	Nanomolar
fM	Femtomolar
pM	Picomolar
