# Correction: Chakraborty et al. A DNA Adsorption-Based Biosensor for Rapid Detection of Ratoon Stunting Disease in Sugarcane. *Biosensors* 2025, *15*, 518

**DOI:** 10.3390/bios15100646

**Published:** 2025-09-30

**Authors:** Moutoshi Chakraborty, Shamsul Arafin Bhuiyan, Simon Strachan, Muhammad J. A. Shiddiky, Nam-Trung Nguyen, Narshone Soda, Rebecca Ford

**Affiliations:** 1 Centre for Planetary Health and Food Security (CPHFS), Nathan Campus, Griffith University, Nathan, QLD 4111, Australia; rebecca.ford@griffith.edu.au; 2 School of Environment and Science (ESC), Nathan Campus, Griffith University, Nathan, QLD 4111, Australia; simon.strachan@griffithuni.edu.au; 3 Queensland Micro- and Nanotechnology Centre (QMNC), Nathan Campus, Griffith University, Nathan, QLD 4111, Australia; sbhuiyan@sugarresearch.com.au (S.A.B.); nam-trung.nguyen@griffith.edu.au (N.-T.N.); n.soda@griffith.edu.au (N.S.); 4 Sugar Research Australia (SRA), 90 Old Cove Road, Woodford, QLD 4514, Australia; 5 Rural Health Research Institute (RHRI), Orange Campus, Charles Sturt University, Orange, NSW 2800, Australia

## Error in Figure

In the original publication [1], there was a mistake in Figure 7A as published. Specifically, the slope, intercept, and efficiency values reported in the regression statistics did not match the plotted data. This discrepancy arose from a transcription error in the original dataset.

The corrected Figure 7A appears below. The recalculated regression statistics are as follows:

Slope (m): −3.215 ± 0.011.
Intercept (b): 39.828 ± 0.037.
Coefficient of determination (R^2^): 0.99996.
Amplification efficiency: 104.7%.

(calculated using E = (10 − 1/m − 1) × 100)

The negative slope is expected for a qPCR standard curve, reflecting the inverse relationship between Cq and log_10_ (copy number). The intercept error in the earlier version (0.04) has been corrected to 39.828, consistent with the regression fit. The recalculated efficiency of 104.7% falls within the MIQE-recommended range of 90–110% for qPCR assays. The R^2^ value of 0.99996 also exceeds the MIQE minimum threshold of 0.99, indicating excellent linearity and reproducibility.

The authors confirm that these corrections do not affect the scientific conclusions of the study. The corrected Figure 7 is provided below with the corrected Figure 7A. This correction was approved by the Academic Editor. The original publication has also been updated.

## Figures and Tables

**Figure 7:**
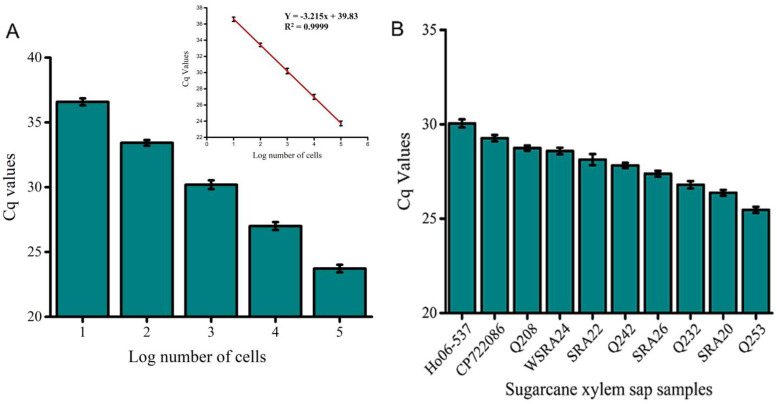
qPCR validation of the electrochemical assay. (**A**) Absolute quantification of *Lxx* DNA in known cell concentrations (10^5^ to 10 cells/μL) confirms the sensitivity of the assay. (**B**) Quantitative detection of *Lxx* in selected field samples infected with RSD. qPCR data show good agreement with EC assay trends. Error bars indicate SD across three independent experiments.
