# Supplementary material for: A DNA Adsorption-Based Biosensor for Rapid Detection of Ratoon Stunting Disease in Sugarcane
Source: Biosensors (Basel). 2025 Aug 8;15(8):518. doi: 10.3390/bios15080518 (PMC12385017; doi:10.3390/bios15080518)
Supplement: Supplementary file 1 [file biosensors-15-00518-s001.zip › biosensors-3765828-supplementary.pdf]

biosensors

MDPI

Supplementary information

A DNA Adsorption-Based Biosensor for Rapid Detection of Ratoon Stunting Disease in Sugarcane

Moutoshi Chakraborty [1,2,*], Shamsul Arafin Bhuiyan [3,4], Simon Strachan [2,3], Muhammad J. A. Shiddiky [5,*], Nam-Trung Nguyen [3], Narshone Soda [3] and Rebecca Ford [1,2]

1 Centre for Planetary Health and Food Security (CPHFS), , Nathan Campus, Griffith University, Nathan, QLD 4111, Australia; rebecca.ford@griffith.edu.au
2 School of Environment and Science (ESC), Nathan Campus, Griffith University, Nathan, QLD 4111, Australia; simon.strachan@griffithuni.edu.au
3 Queensland Micro- and Nanotechnology Centre (QMNC), , Nathan Campus, Griffith University, Nathan, QLD 4111, Australia; sbhuiyan@sugarresearch.com.au (S.A.B.); nam-trung.nguyen@griffith.edu.au (N.-T.N.); n.soda@griffith.edu.au (N.S.)
4 Sugar Research Australia (SRA), 90 Old Cove Road, Woodford, Qld 4514, Australia
5 Rural Health Research Institute (RHRI), Orange Campus, Charles Sturt University, Orange, NSW 2800, Australia
* Correspondence: m.chakraborty@griffith.edu.au (M.C.); mshiddiky@csu.edu.au (M.J.A.S.)

Supplementary Table and Figures

Table S1. Details of the probe and primer sets designed for developing *Lxx*-specific EC and qPCR assay.

Synthetic Target and Primers Name	Sequence (5′-3′)	Length (nt)	GC (%)
*Lxx*_EC_STS	GCTCGAACTTAGTACGCCTGCTTGCAGGAAGGAACAGTTCGGACCGGGGAGCCTCGCACATGCACGCTGTTGGGTCCTGAGGGACCGGACCTCATCGCTGTGTCTTCAAGACGCTGAGATGAGAACCGAATCC	133	58.6
*Lxx*CP1	GGATTCGGTTCTCATCTCAGCGTCTTGAAGACAC/Bio/	34	50
*Lxx*_EC_FP	GCTCGAACTTAGTACGCCTG	20	55
*Lxx*_EC_RP	GGATTCGGTTCTCATCTC	18	50

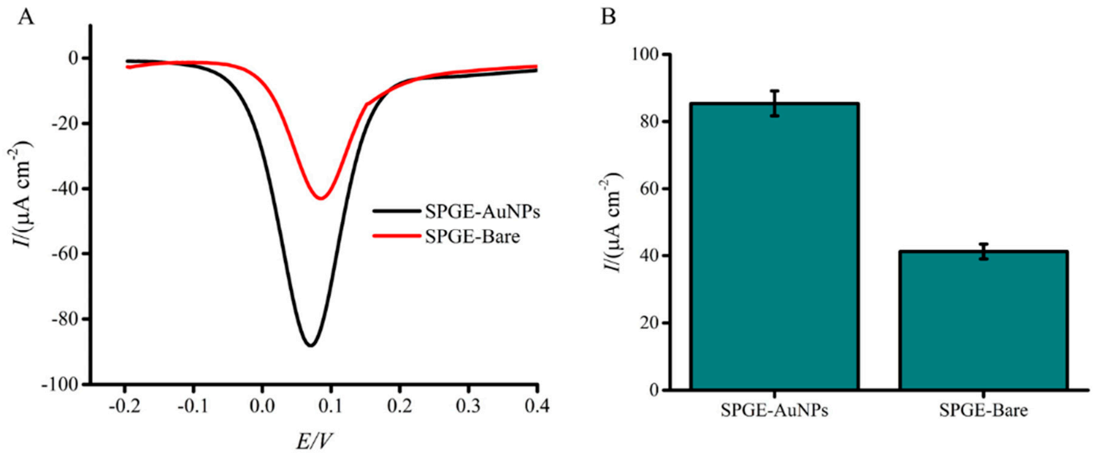

Figure S1. Optimization of AuNP adsorption on SPGEs. (A) DPVs comparing the electrochemical response of a bare electrode (without AuNPs) and an AuNP-modified electrode. (B) Average

 http://doi.org/10.3390/bios15080518

percentage change in current response between the bare and AuNP-modified electrodes, indicating enhanced signal upon AuNP modification. Error bars represent the standard deviation from three independent measurements.

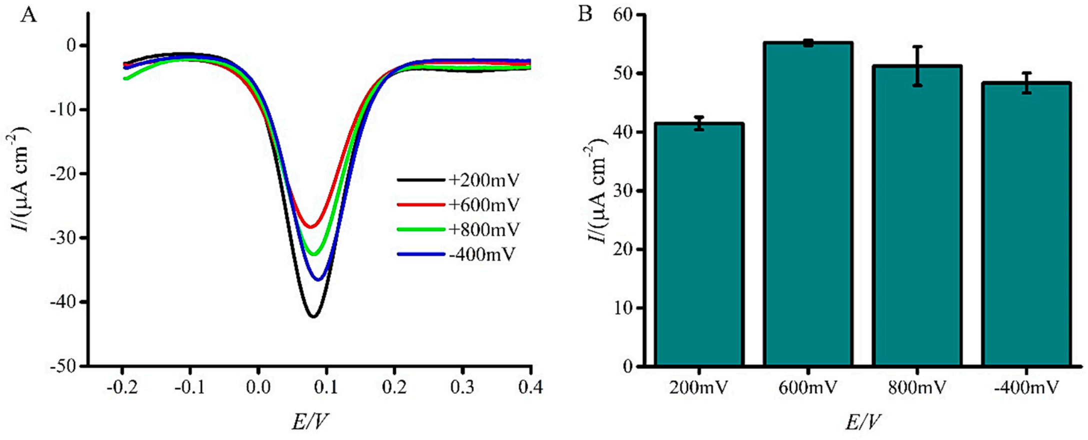

Figure S2. Optimization of the adsorption potential for DNA immobilization on gold-modified electrodes. (A) DPVs showing the electrochemical response of electrodes following DNA adsorption at four different potentials: +200 mV, +600 mV, +800 mV, and –400 mV (vs. Ag/AgCl). (B) Average percentage change in current response corresponding to each adsorption potential, highlighting the influence of potential on DNA-gold interaction efficiency. Error bars represent the standard deviation from three independent experiments.

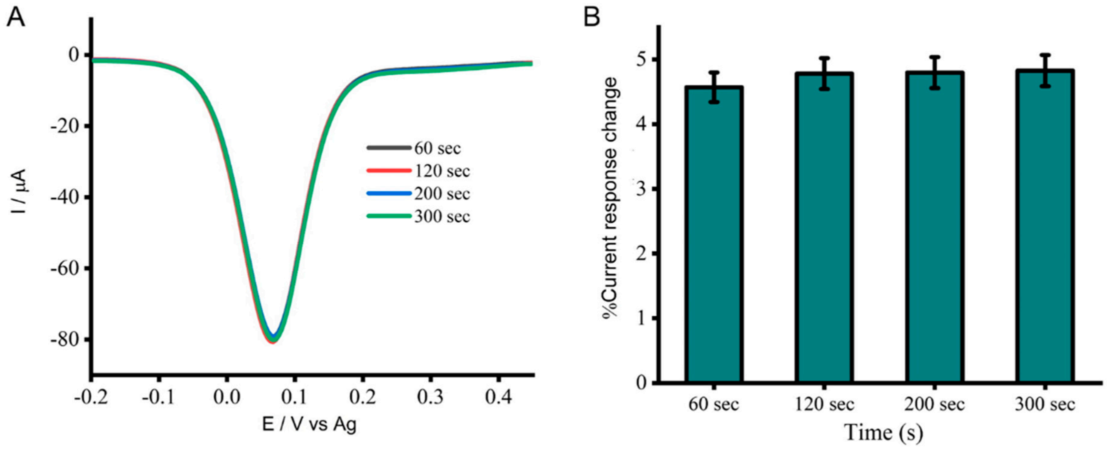

Figure S3. Optimization of DNA adsorption time on gold-modified electrodes.
(A) DPVs recorded after DNA immobilization on gold-modified electrodes for varying durations: 60, 120, 200, and 300 seconds. (B) Average percentage change in current response corresponding to each adsorption time, illustrating the effect of incubation time on DNA-gold binding efficiency. Error bars represent the standard deviation from three independent experiments.

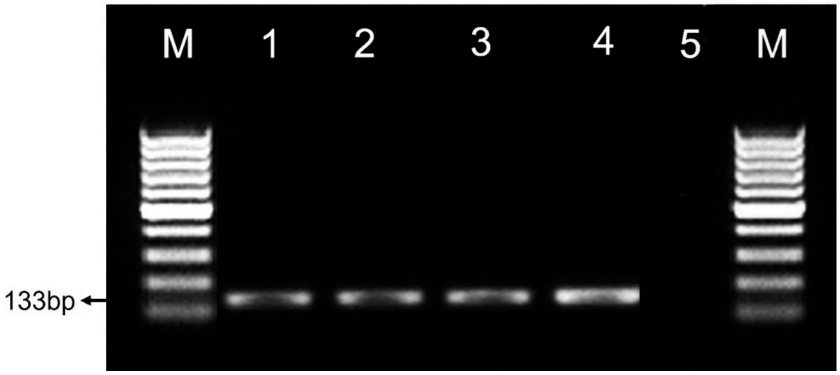

Figure S4. Agarose gel electrophoresis of qPCR-amplified products from varying concentrations of *Lxx* cells. Lane M: 100 bp DNA ladder (marker); Lanes 1–4: qPCR products obtained from samples containing 10^2 to 10^5 Lxx cells/μL, respectively; Lane 5: no target control (NTC), showing absence of amplification in the negative control.

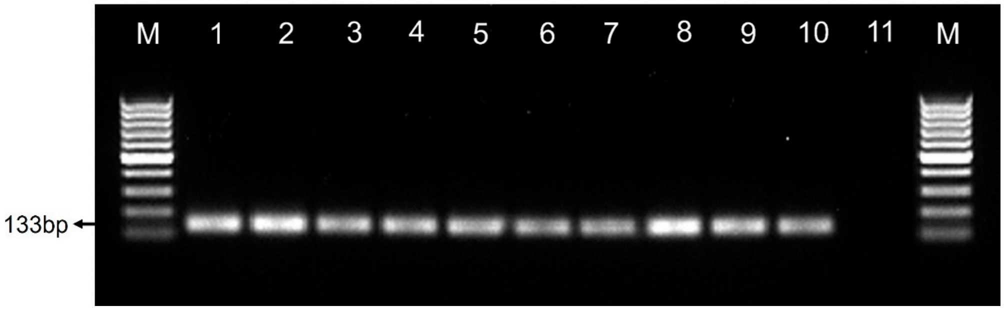

Figure S5. Detection of Lxx in ratoon-stunting disease-infected sugarcane samples using qPCR and agarose gel electrophoresis. Lane M: 100 bp DNA ladder (marker); Lanes 1–10: qPCR-amplified products from xylem sap of RSD-infected sugarcane cultivars—Ho06-537, CP72-2086, Q208, WSRA24, SRA22, Q242, SRA26, Q232, SRA20, and Q253, respectively; Lane 11: no target control (NTC), confirming the absence of non-specific amplification.
